# Human Pulse Detection by a Soft Tactile Actuator

**DOI:** 10.3390/s22135047

**Published:** 2022-07-05

**Authors:** Zixin Huang, Xinpeng Li, Jiarun Wang, Yi Zhang, Jingfu Mei

**Affiliations:** 1 School of Electrical and Information Engineering, Wuhan Institute of Technology, Wuhan 430205, China; lixinpeng@stu.wit.edu.cn (X.L.); wangjiarun@stu.wit.edu.cn (J.W.); zhangyi@stu.wit.edu.cn (Y.Z.); meijingfu@stu.wit.edu.cn (J.M.); 2 Hubei Key Laboratory of Digital Textile Equipment, Wuhan Textile University, Wuhan 430200, China; 3 Key Laboratory of Textile Fiber and Products, Wuhan Textile University, Ministry of Education, Wuhan 430200, China; 4 State Key Laboratory of New Textile Materials and Advanced Processing Technologies, Wuhan Textile University, Wuhan 430200, China

**Keywords:** soft sensing technology, soft tactile actuator, pulse detecting algorithm

## Abstract

Soft sensing technologies offer promising prospects in the fields of soft robots, wearable devices, and biomedical instruments. However, the structural design, fabrication process, and sensing algorithm design of the soft devices confront great difficulties. In this paper, a soft tactile actuator (STA) with both the actuation function and sensing function is presented. The tactile physiotherapy finger of the STA was fabricated by a fluid silica gel material. Before pulse detection, the tactile physiotherapy finger was actuated to the detection position by injecting compressed air into its chamber. The pulse detecting algorithm, which realized the pulse detection function of the STA, is presented. Finally, in actual pulse detection experiments, the pulse values of the volunteers detected by using the STA and by employing a professional pulse meter were close, which illustrates the effectiveness of the pulse detecting algorithm of the STA.

## 1. Introduction

In recent years, the development of robotics has been impacted by innovative research on natural creatures and biological processes [1]. Instead of rigid materials, the trend is to use soft materials to fabricate soft structures for soft robots [2]. Soft robots have the advantages of high structural flexibility [3], good environmental adaptability [4], strong human–computer interaction [5], and high safety [6]. With the developments in soft actuation technology and soft sensing technology, soft robots are expanding into human–computer interaction, health care, field exploration, and other fields [7,8]. Moreover, with the increasing maturity of the soft material processing technology, various soft robots have been fabricated, such as the soft crawl robot [9], soft underwater robot [10], and soft flying robot [11].

Health care is a significant application field for soft robots [12]. Soft robots can be designed as wearable devices, which can be used to help with the physiological activities of people through their actuation functions. However, in previous studies and current applications, the physiological data of humans have only been detected by using professional external sensors. With the development of soft sensing technologies, soft sensors exhibit a wide range of application prospects. In particular, by combining the soft actuation technology and the soft sensing technology, soft robots can assist in the rehabilitation training of patients, without bringing any discomfort and injury to the body, and detect human physiological signals.

The pneumatic soft robot based on pneumatic actuation technology is an important branch of soft robots [13]. The fabrication process of the pneumatic soft robot is more mature than that of soft robots based on other soft actuation technologies, which is conducive to rapid engineering applications [14]. The pneumatic soft actuator is an essential part of the soft robot. Previous studies of a pneumatic soft robot have mainly focused on the structural design and actuation function implementations of the pneumatic soft actuator [15,16]. In [17], a spine-inspired bistable soft actuator was designed to fabricate a cheetah-like soft robot. In [18], four pneumatic soft actuators were employed to design a soft-legged untethered quadruped robot. In [19], a fully soft bionic grasping device based on pneumatic actuation technology was used to grab objects of different shapes, such as bottles, balls, boxes, and so on. However, the aforementioned pneumatic soft actuators only focused on the actuation function, while their sensing function was not explored. Inspired by this idea, we developed a soft tactile actuator (STA) based on the pneumatic soft actuator, which is a new concept of the soft device with both an actuation function and a sensing function. Obviously, the structural design of the STA confronts great challenges.

The sensing algorithm of the STA is a key part to realize its sensing function. However, the sensing algorithm of the STA has rarely been studied. In previous studies, there have been some soft sensing technologies explored on soft smart materials. In [20], a strain sensor based on an ionic-hydrogel was fabricated, and a series of experiments were conducted to test the influences of different external factors on its sensing performance. In [21], a soft force and displacement sensor was fabricated based on dielectric elastomer material, and a sensing model was established to describe the sensing properties. However, these studies only focused on the sensing functions of the smart material and did not combine the sensing function with practical applications. In addition, there is a lack of relevant studies on the actuation function and the sensing function of the STA simultaneously. Therefore, there are great difficulties in the structural design, actuation modeling, and sensing algorithm design of STA.

In this work, we designed an STA with both the actuation function and the sensing function. The STA is composed of a base plate, two tactile physiotherapy fingers, actuation pipes, and a tactile stress sensor. Then, the position teaching of the STA was conducted to acquire the air pressures that could actuate the finger to reach the premeasurement status. Next, a sensing algorithm of the STA was designed to implement its sensing function to acquire the pulse signal of a human. Finally, some experiments were conducted to verify the effectiveness of the pulse detecting algorithm of the STA.

## 2. System Description

In this section, the fabrication flow of the STA and the construction of the experimental platform are stated briefly.

### 2.1. Fabrication Flow

**Figure 1:**
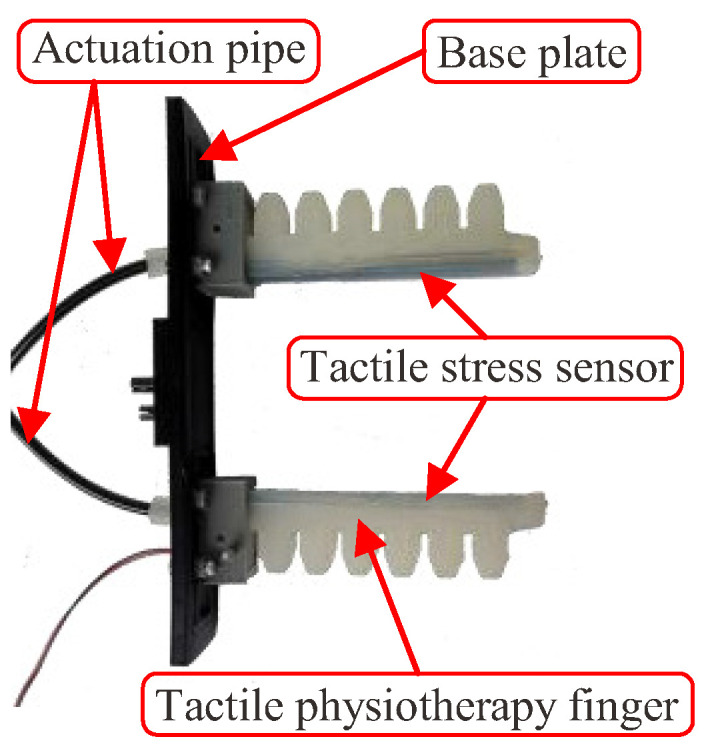
Finished product of the STA.

As shown in Figure 1, the STA was composed of a base plate, a tactile physiotherapy finger, an actuation pipe, and a tactile stress sensor. The actuation pipe and the tactile stress sensor were assembled on the tactile physiotherapy finger. Then, two tactile physiotherapy fingers with the actuation pipe and the tactile stress sensor were mounted on the base plate.

**Figure 2:**
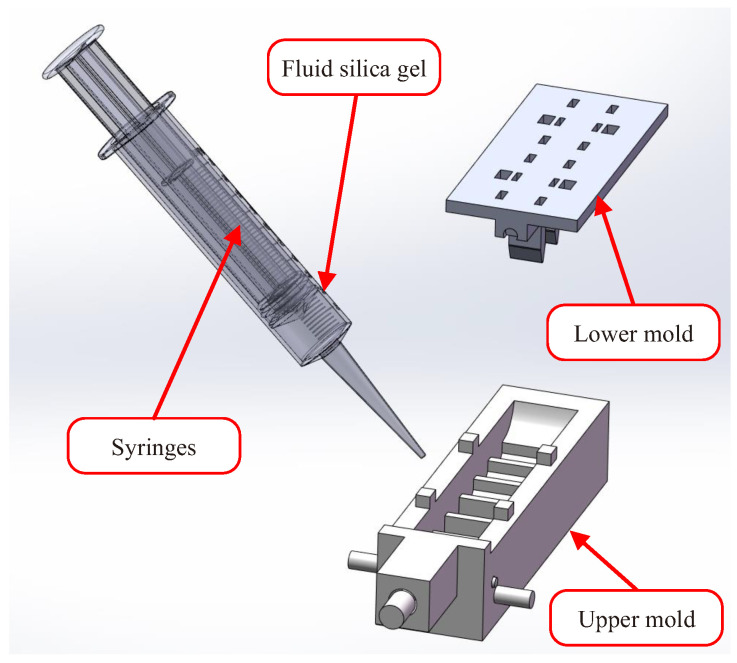
Injection molding process of the upper mold.

**Figure 3:**
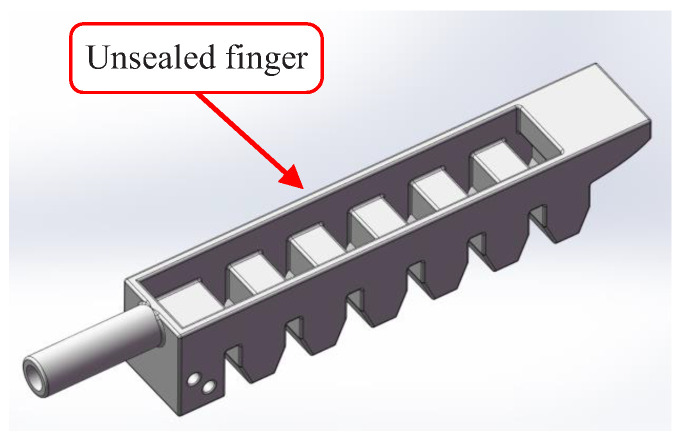
Unsealed finger.

**Figure 4:**
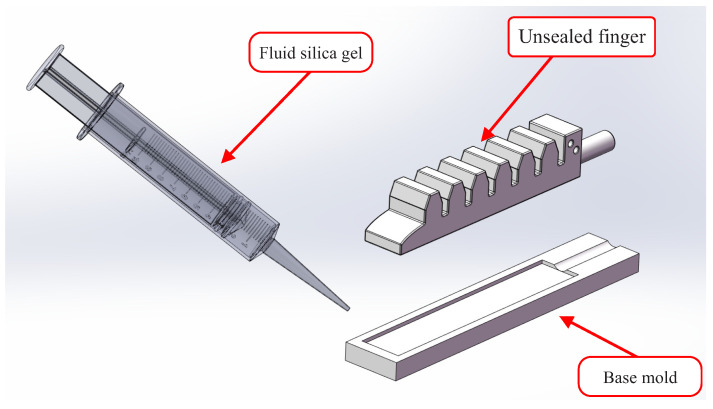
Injection molding process of the base mold.

**Figure 5:**
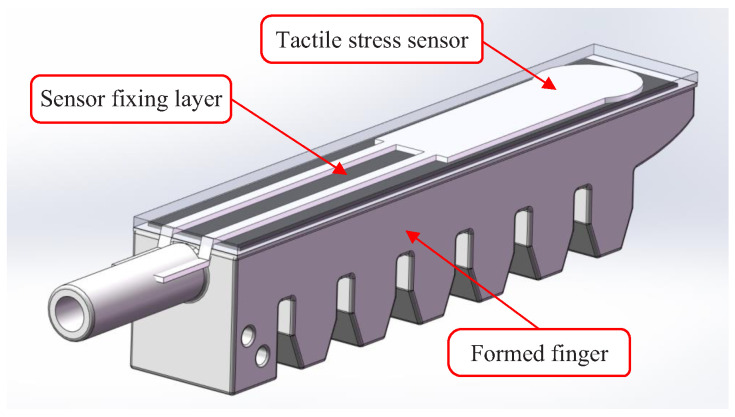
Tactile physiotherapy finger.

The most complex component of the STA was the tactile physiotherapy finger. The manufacturing flow of the tactile physiotherapy finger was as follows. First, as shown in Figure 2, the molds of the tactile physiotherapy finger were fabricated in advance. Then, the fluid silica gel was cast into the upper mold by using the syringes, and the lower mold was clamped into the upper mold. After the fluid silica gel was cured, the unsealed finger (as shown in Figure 3) was formed. Next, as shown in Figure 4, the fluid silica gel was cast into the base mold by using the syringes. After the fluid silica gel was cured, the formed finger was obtained. Finally, as shown in Figure 5, the tactile stress sensor was fixed on the formed finger using the cured silica gel as the sensor fixing layer to fabricate the tactile physiotherapy finger.

**Remark**: Injection molding is a simple and effective method to fabricate various pneumatic devices [19]. In the above manufacturing of the tactile physiotherapy finger, injection molding was employed. The fluid silica gel material is easily employed in injection molding, and it can ensure the softness property of the tactile physiotherapy finger. So, the fluid silica gel material was employed in this paper. Moreover, to improve the quality of the tactile physiotherapy finger, a mold with a smooth surface should be employed. We fabricated several tactile physiotherapy fingers at the same time. During the injection molding process, we placed the molds filled with the fluid silica gel into the baker to ensure that the fluid silica gel was cured at a constant temperature. By this means, we obtained the fingers without cracks or bubbles. After the injection molding process, we inspected the appearance and size for each finger and tested its deformation performance by applying compressed air to the chamber. By comprehensively considering the above requirements, we selected a tactile physiotherapy finger to fabricate the STA.

### 2.2. Experimental Platform

**Figure 6:**
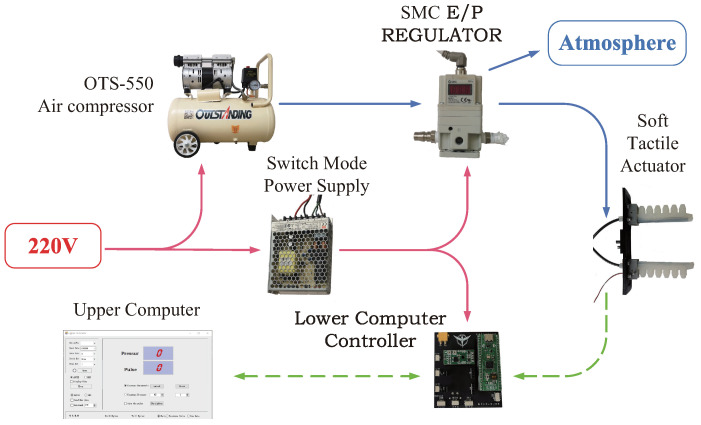
Experimental platform of the STA.

In order to study the actuation and tactile functions of the STA, an experimental platform (see Figure 6) was constructed. The power supply of the whole system was 220 (V), which was directly employed to power the air compressor (Manufacturer: OUTSTANDING; Model: OTS-550) and used as the input of the switch mode power supply (Manufacturer: TAIWEI; Model: LRS-100-24). The air compressor was exploited to supply the compressed air for the whole system. The proportional pressure regulating valve (Manufacturer: SMC Corporation; Model: ITV2050-312L) was used to regulate the air pressure. The STA was actuated by the compressed air supplied from the actuation pipe. Meanwhile, the pulse detecting function of the STA was realized by detecting the stress imposed on the tactile stress sensor (Manufacturer: K-CUT; Model: RP-C7.6-LT-LF2). The stress data were acquired and processed by the lower computer controller. Finally, the upper computer was designed to display the stress detected by using the tactile stress sensor and the pulse of the human body.

## 3. Detecting Position Training of the STA

Before starting the pulse detection, the finger of the STA should be move to the premeasurement position. In actual pulse detection, high-precision position control of the STA is not required to implement the pulse detection function. So, in this section, we describe how we conducted the position training of the STA to acquire the air pressures that could actuate the finger to the detecting positions.

### 3.1. Regulation of Compressed Air

As shown in Figure 6, the air pressure used to actuate the finger of the STA was regulated by the proportional pressure regulating valve. Moreover, pulse width modulation (PWM) technology was employed in the proportional pressure regulating valve to control the air pressure. The output air pressure of the proportional pressure regulating valve was

(1) $$P=\alpha P_{\max}$$

where α is the duty ratio of the PWM, and $P_{\max}$ is the maximum output air pressure of the proportional pressure regulating valve.

### 3.2. Detecting Position Training

In actual pulse detection, high-precision position control of the STA is not required. We only need to move the tactile physiotherapy finger of the STA to the premeasurement position before starting the pulse detection. This goal can be easily realized by training the STA.

Through repeatedly conducting actuation experiments on the STA and observing the deformation of its tactile physiotherapy finger, we set the range of $P_{\max}$ as 0.09 to 0.25 (MPa). Meanwhile, α=0.4 was applied in detecting process. By setting different values for $P_{\max}$, compressed air with different air pressures can be injected into the tactile physiotherapy finger of the STA. Correspondingly, the displacement *D* of the tactile physiotherapy finger is shown in Table 1.

**Figure 7:**
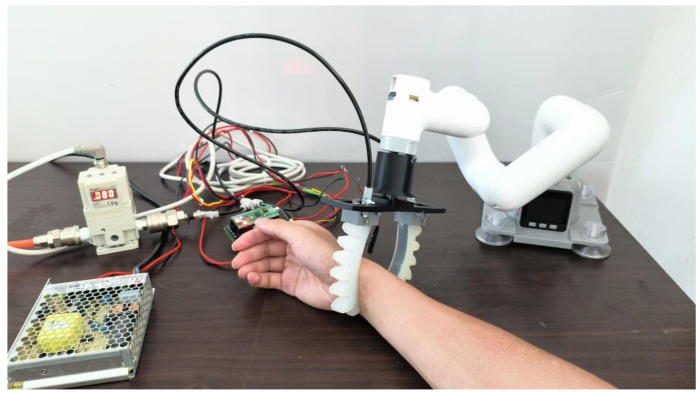
Picture of pulse detection with the STA.

According to Table 1, we can estimate the air pressure according to the desired displacement of the STA. When P=0.080 (MPa) was used to act in the STA, its tactile physiotherapy finger could grab the wrist of a person, making the tactile stress sensor reliably contact with the detection position (as shown in Figure 7). Then, we employed the STA to detect the pulse. In the practical detection process, the air pressure *P* can be slightly adjusted according to the arm size of different people. It is precisely due to the application of soft actuation technology and soft sensing technology that STA has a good adaptability and high safety.

## 4. Tactile Sensing Scheme of STA

In this section, the pulse detecting mechanism is explained first. Then, the tactile sensing algorithm’s development is presented.

### 4.1. Pulse Detecting Mechanism

According to the results presented in Section 3, when applying compressed air with the pressure of 0.080 (MPa) to the STA, its tactile physiotherapy finger can grab the arm of human, bringing the tactile stress sensor close to the point to be detected. If the tactile stress sensor is not subjected to external force, the stress detected by using the tactile sensor is constant. A human pulse imposes a periodically variable external force on the tactile stress sensor, which leads to the stress detected by using the tactile senor to also vary periodically. According to the detected stress, the pulse can be computed.

### 4.2. Tactile Sensing Algorithm

In this subsection, the tactile sensing algorithm to process the detected stress data and eventually achieve pulse detection is presented.

**Figure 8:**
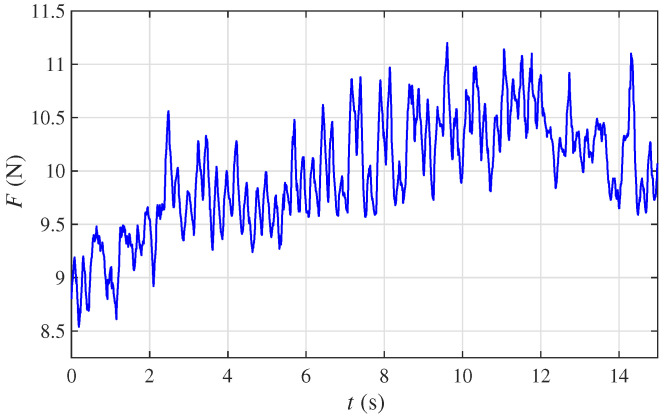
Stress data of volunteer A.

Without losing generality, a set of stress data of a volunteer (denoted as volunteer A) was detected by the tactile stress sensor, which is shown in Figure 8. The total time was $t_{d}=15$ (s), and the sampling time was h=0.01 (s). As can be seen visually from Figure 8, the detected data were seriously impacted by the noise. So, we first processed the detected data to reduce the influence of the noise.

For ease of computing the pulse value, the detected stress data were converted to binary sequences. At the *k*-th sampling, the stress was denoted as $F\left(k\right)$. The variation of the stress was defined as

(2) $$\Delta F\left(k\right)=\left\{\begin{array}{c} F\left(1\right)-0=F\left(1\right),\;k=1\\ F\left(k+1\right)-F\left(k\right),\;k⩾2 \end{array}\right.$$

The converting rule was as follows

(3) $$F_{B}\left(k\right)=\left\{\begin{array}{c} 1,\;if\;\Delta F>0\\ 0,\;if\;\Delta F⩽0 \end{array}\right.$$

where $F_{B}\left(k\right)$ is the converted binary value of the *k*-sampling data.

To remove the outliers, a sliding window with a length of 4 was employed to filter the binary sequences $F_{B}$. For ease of illustration, the data selected by the window were denoted as $a_{1}$, $a_{2}$, $a_{3}$ and $a_{4}$, respectively. The window slid one grid at a time and generated the filtered data. Based on (3), the filter rule was as follows

(4) $$F_{BF}\left(k\right)=\left\{\begin{array}{c} 1\;\sum_{i=k,\;k=1,2,\ldots ,N-3}^{i+3}F_{B}\left(i\right)=4\\ 0,\;\sum_{i=k,\;k=1,2,\ldots ,N-3}^{i+3}F_{B}\left(i\right)=0\\ F_{B}\left(k\right),\sum_{i=k,\;k=1,2,\ldots ,N-3}^{i+3}F_{B}\left(i\right)\notin \left[\begin{array}{cc} 0 & 4 \end{array}\right] \end{array}\right.$$

where *N* was the total number of the binary sequences $F_{B}$.

To count the number of pulses, according to (4), a new sequence was generated

(5) $$F_{BP}\left(k\right)=\left\{\begin{array}{c} 1,F_{BF}\left(k+1\right)-F_{BF}\left(k\right)=1\\ 0,F_{BF}\left(k+1\right)-F_{BF}\left(k\right)\neq 1 \end{array}\right.$$

According to (5), the number of pulses within 1 min was

(6) $$N_{d}=\frac{60}{t_{d}}\sum_{k=1}^{N-3}F_{BF}\left(k\right)$$

In fact, when selecting a small value for $t_{d}$, the detection speed is fast, but the detection precision may be not good. On the other hand, when selecting a large value for $t_{d}$, the detection precision is ensured, but the detection speed is slow. So, there is a tradeoff between the detection precision and the detection speed. Through repeated experiments, we set $t_{d}=15$ (s).

To avoid the influence of accidental factors on the detection results, the average value of the multiple measurements was employed as the pulse, which was

(7) $$N_{p}=\frac{1}{5}\sum_{i=1}^{5}N_{d_{i}}$$

where $N_{d_{i}}$ was the *i*-th detecting value.

## 5. Pulse Detection Experiment

The pulse detecting function of the STA was verified by conducting several experiments.

As shown in Figure 8, the radial artery of the human was attached by the tactile pipe of the STA. According to the detection method presented in Section 4.2, the pulse was detected. To verify the validity of the pulse detecting function of the STA, the pulses of five volunteers were detected by employing the STA and the professional pulse meter (Manufacturer: GUANCHANG; Model: BSX526), respectively. For ease of statement, these five volunteers were denoted as A, B, C, D, and E, respectively.

For volunteer A, the stress data detected by the tactile stress sensor are shown in Figure 8. To describe the process of the tactile sensing algorithm clearly, we selected a subset of the detected data to explain how the tactile sensing algorithm worked.

**Figure 9:**
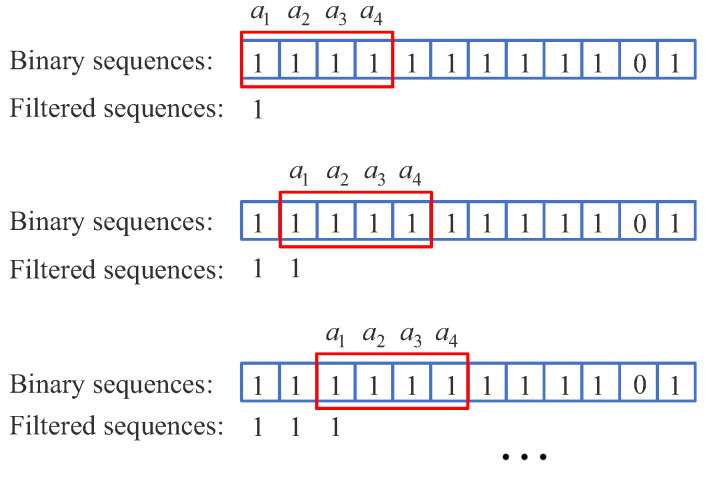
Filtering process using sliding window.

**Figure 10:**
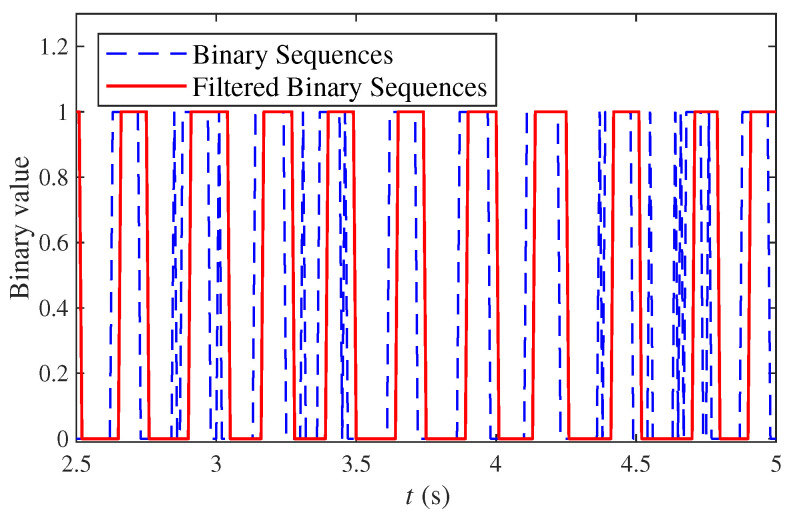
Comparison of the binary sequences and the filtered binary sequences within $t\in \left[2.5,5.0\right]$.

According to (3) and (4), the filtering process using the sliding window is shown in Figure 9. To clearly show the filtering effect, the comparison of the binary sequences $F_{B}$ and the filtered binary sequences $F_{BF}$ within $t\in \left[2.5,5.0\right]$ (s) is shown in Figure 10. By repeating the above process, the filtered binary sequences were obtained. According to (6), $N_{d}=66$. Through conducting multiple measurements, the detection results are shown in Table 2. According to (7), the pulse of volunteer A was $N_{p}=65$. Moreover, the pulse of volunteer A detected by the professional pulse meter was $N_{I}=65$. By comparing these two results, the detection result of the STA using the tactile sensing algorithm was effective and credible.

To further verify the effectiveness and generality of the proposed tactile sensing algorithm, the pulses of volunteers B, C, D, and E were conducted. The detected results are shown in Table 2. According to Table 2 and Equation (6), the pulse of the volunteers computed by using the proposed tactile sensing algorithm are shown in Table 3. Moreover, the comparison of the pulse values of volunteers detected by using STA and that by employing professional pulse meter is also shown in Table 3. From Table 3, it is easy to see that the pulse values detected by using the STA were close to those of the professional pulse meter, which illustrates the effectiveness of the proposed tactile sensing algorithm.

According to the above experiments, the pulse detecting function of the STA was realized. Moreover, for each volunteer, $N_{p}$ and $N_{I}$ were close, which means that the detection result of the STA was accurate and reliable; further, the proposed tactile sensing algorithm was effective. In addition, we selected five different volunteers to conduct the pulse detection experiments and the detection results of the STA were close to that of the professional pulse meter, which illustrates that the proposed tactile sensing algorithm has good generalization performance.

## 6. Conclusions

In this paper, a soft tactile actuator was designed and fabricated. Then, to move the tactile physiotherapy finger of the STA to the detecting position, the position training of the STA was conducted. Next, the pulse detecting algorithm was presented to realize the pulse detection function of the STA. Finally, several detection experiments were conducted. In each experiment, the pulse detection result of the STA was close to that of the professional pulse meter, which illustrates that the proposed pulse detecting algorithm is effective. Furthermore, we selected five different volunteers to conduct the pulse detection experiments, and the STA accurately detected the pulse of each volunteer, which illustrates that the proposed tactile sensing algorithm has good generalization performance.

Compared to an existing pulse meter, the STA can be used as a senor to detect a pulse and can also be employed as an actuator to conduct massage and physiotherapy. Moreover, since the STA is soft and flexible, it can safely interact with a human. Therefore, the STA has broad application prospects in the fields of health care and intelligent care of the elderly. However, since the STA is a new type of sensor, there is not a specification for its calibration. So, in our future work, we plan to study the calibration of the STA and formulate specifications to pave the way for its application in the fields of health care and intelligent care of the elderly.

## Figures and Tables

**Table 1:** Position training results of the STA.

$P_{\max}$ (MPa)	*P* (MPa)	*D* (cm)
0.09	0.036	0.90
0.10	0.040	2.50
0.11	0.044	2.35
0.12	0.048	2.55
0.13	0.052	2.90
0.14	0.056	3.10
0.15	0.060	3.40
0.16	0.064	3.50
0.17	0.068	3.75
0.18	0.072	4.15
0.19	0.076	4.40
0.20	0.080	4.60
0.21	0.084	4.90
0.22	0.088	5.20
0.23	0.092	5.30
0.24	0.096	5.55
0.25	0.010	5.90

**Table 2:** Pulse detection results $N_{d}$ of the volunteers.

*i*	A	B	C	D	E
1	66	66	74	75	72
2	65	66	74	78	71
3	66	67	73	80	74
4	63	63	74	76	74
5	64	69	72	78	75

**Table 3:** Comparison of the pulse values of the volunteers detected by using STA and by employing a professional pulse meter.

Volunteer	$N_{p}$	$N_{I}$
A	65	65
B	66	68
C	74	74
D	77	78
E	74	75

## Data Availability

Not applicable.
